# Conformational and thermodynamic hallmarks of DNA operator site specificity in the copper sensitive operon repressor from *Streptomyces lividans*

**DOI:** 10.1093/nar/gkt902

**Published:** 2013-10-08

**Authors:** Benedict G. Tan, Erik Vijgenboom, Jonathan A. R. Worrall

**Affiliations:** ^1^School of Biological Science, University of Essex, Wivenhoe Park, Colchester, CO4 3SQ, UK and ^2^Molecular Biotechnology, Institute of Biology Leiden, Sylvius Laboratory, Leiden University, PO Box 9505, 2300 RA Leiden, The Netherlands

## Abstract

Metal ion homeostasis in bacteria relies on metalloregulatory proteins to upregulate metal resistance genes and enable the organism to preclude metal toxicity. The copper sensitive operon repressor (CsoR) family is widely distributed in bacteria and controls the expression of copper efflux systems. CsoR operator sites consist of G-tract containing pseudopalindromes of which the mechanism of operator binding is poorly understood. Here, we use a structurally characterized CsoR from *Streptomyces lividans* (CsoR^Sl^) together with three specific operator targets to reveal the salient features pertaining to the mechanism of DNA binding. We reveal that CsoR^Sl^ binds to its operator site through a 2-fold axis of symmetry centred on a conserved 5′-TAC/GTA-3′ inverted repeat. Operator recognition is stringently dependent not only on electropositive residues but also on a conserved polar glutamine residue. Thermodynamic and circular dichroic signatures of the CsoR^Sl^–DNA interaction suggest selectivity towards the A-DNA-like topology of the G-tracts at the operator site. Such properties are enhanced on protein binding thus enabling the symmetrical binding of two CsoR^Sl^ tetramers. Finally, differential binding modes may exist in operator sites having more than one 5′-TAC/GTA-3′ inverted repeat with implications *in vivo* for a mechanism of modular control.

## INTRODUCTION

**Figure 1.**
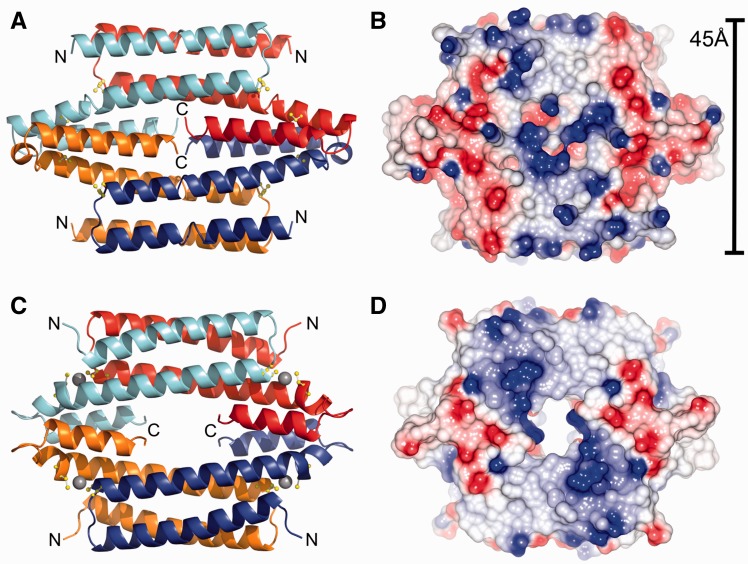
Structural characteristics of the apo-form of CsoR from *Streptomyces lividans* (top) (PDB 4adz) (11) and the Cu(I)-bound form from *Mycobacterium tuberculosis* (bottom) (PDB 2hh7) (7). (**A**) and (**C**) show a cartoon view of the protomer arrangement in the tetrameric organization of CsoR. The Cys residues that constitute the inter-protomer Cu(I) binding site are shown in ball and stick, and the bound Cu(I) ion in the structure of CsoR^Mtb^ are represented as a grey spheres. (**B**) and (**D**) illustrates the electropositive surface potential of the tetrameric surface of both CsoR proteins, which is flanked by negatively charged domains around the Cu(I)-binding sites. The width of the tetrameric surface for CsoR^Sl^ is indicated.

In bacteria, families of metal sensing transcriptional regulators, commonly referred to as metalloregulatory or metal sensor proteins, act to control the expression of genes that allow the organism to quickly adapt to chronic toxicity or deprivation of biologically essential metal ions (1–4). These proteins are able to form specific metal ion coordination complexes, with metal affinities as high as femto- to zeptomolar for Cu(I) and some Zn(II) sensors (5,6), and can either inhibit or activate operator DNA binding or directly enhance transcriptional activation (2). At present, seven structural metalloregulatory families have been identified and characterized to varying extents (2). The CsoR/RcnR family has members which are known to directly respond to Cu(I) ions (CsoR) (7–12), Ni(II)/Co(II) ions (RcnR) (13,14), Ni(II) ions (InrS) (15) or to inorganic sulphur (CstR) (12,16). Structurally, only the copper sensitive operon repressor (CsoR) proteins from *Mycobacterium tuberculosis* (7), *Thermus thermophilus* (10) and more recently from *Streptomyces lividans* (11) have been determined and all in the absence of operator DNA. These CsoR proteins exist in solution as tetrameric assemblies with each protomer consisting of three α-helices of varying lengths (Figure 1). Interactions between the α3 helices of each protomer are important for maintaining the tetramer assembly. The significance, if any, of the ‘hole’ present in the *M. tuberculosis* CsoR model (Figure 1) is not known, but it is noted that a long C-terminal tail, absent in *S. lividans* CsoR, is not observed in the *M. tuberculosis* structure. An inter-subunit Cu(I) binding site is formed between two Cys thiolates and the Nδ2 atom of a His residue. One coordinating Cys and His residue are positioned towards the C-terminal end of the α2 helix, and the second Cys ligand is located at the N-terminal end of the α2′ helix of a second protomer (Figure 1).

CsoR binds to its DNA operator in the apo-state with a 2:1 CsoR tetramer:DNA stoichiometry (7,9,11,12). Under Cu stress conditions, Cu(I) ions bind to the DNA bound apo-CsoR with attomolar (10^−^^18 ^M) affinity and allosterically activate transcriptional derepression (7). Compared with other metalloregulator families, the α-helical disc-shaped structure of CsoR is unique for its absence of a recognizable DNA binding domain, e.g. winged helix or ribbon-helix-helix (2). The helices in these domains are known to establish several types of interactions with DNA, predominantly at the major groove where functional groups of base pairs are most exposed. Direct (base-specific) readout mechanisms in these domains are part of a recognition helix, which is often stabilized by indirect (shape-specific) readout or non-specific interactions from polar/charged residues around the interface (17). Although the CsoR assembly is tetrameric in solution, each tetrameric face is essentially dimeric, having two complete monomeric elements (α1-α2-α3′ helices) per tetrameric face (Figure 1). An electropositive surface area runs diagonally (northwest to southeast) across these antiparallel CsoR dimers (Figure 1) implying that contact with one face of its DNA operator by the electropositive tract can be achieved by spanning the α1→α1′ components, where the 2:1 CsoR:DNA stoichiometry can be thought of as establishing a ‘wrapping’ around both faces of the DNA site. Experimental evidence using ratiometric pulse-chase amidination mass spectrometry as a means to monitor the reactivity of lysine residues of *Bacillus subtilis* CsoR in complex with its DNA operator has provided good experimental support for the DNA operator making contact with the surface electropositive tract of CsoR (18).

Known and predicted operator sequences for the CsoR/RcnR family can be grouped into two distinct types of sites (19). Type 1 sites have a single G/C-tract of between three to eight bases flanked by AT-rich inverted repeats of varying length, whereas the type 2 sites have two shorter G/C tracts with two to four intervening bases and the AT-rich inverted repeat outside of the two G/C tracts. The spacing between inverted AT-rich repeats in type 2 sites is typically 11 base pairs and for type 1 sites six to nine base pairs. In addition, tandem sites in certain organisms have been identified, which consist of two separate sites of type 1 or type 2 or both. The Ni(II)/Co(II) RcnR metalloregulator from *Escherichia coli* recognizes a tandem operator site consisting of two type 1 sites with a TACT-G_6_-N-AGTA sequence and is reported to bind to the minor grooves of the TACT/AGTA inverted repeats at both ends of each type 1 site with a 1:1 stoichiometry (19). The binding mechanism for RcnR suggests features of shape selective or indirect readout-based recognition for its operator sequence as well as specific features of the flanking base pairs. This shape selectivity is attributed to the unique conformation brought about by unbroken G/C-tracts in the type 1 operator site, which are considered to endow A-form DNA characteristics (19). For CsoR members, the known DNA operator sites fall into the type 2 category, with semi-continuous G/C-tracts with two tetramers binding at this site as opposed to one RcnR at the type 1 site. Knowledge of how CsoR proteins recognize and bind their type 2 DNA operator site is limited to the identification of the electropositive tract on CsoR and from a CsoR paralogue, CstR, where shape selectivity towards the G/C-tracts has been implied (12,18).

In the antibiotic producing bacteria streptomycetes, genes for CsoR proteins and paralogues have been identified (20). Certain *Streptomyces* strains show a distinct dependence on the bioavailability of Cu ions for their morphological development (21–24). Cuproproteins and enzymes have been revealed to have a key role in initiating a development switch in the life cycle (24,25), with CsoR acting to maintain cytosolic Cu homeostasis (11). The recently reported genome sequence of *Streptomyces lividans* 1326 has identified two genes that encode for Cu(I)-CsoR proteins (26). Before this genome information, a previous study had genetically, biochemically and structurally characterized the *csoR-SLI4375* gene from *S. lividans* 1326 (11). Three regulons under the control of CsoR-4375 (CsoR^Sl^) were identified with all DNA operator sites being classified as type 2 sites (11). The presence of a second CsoR protein suggests the possibility that two independent Cu sensitive regulons may be operational in *S. lividans* 1326. In the present study, we have used a combination of protein and DNA mutations to explore the interplay between protein and DNA elements associated with the specificity of the CsoR^Sl^ DNA operator interaction. An ensemble of computational and biophysical techniques has yielded results that highlight a number of conformational and thermodynamic features pertaining to the localization of the CsoR^Sl^ oligomeric complex at the operator site. Our findings suggest that CsoR^Sl^ facilitates high-affinity contacts to the terminal ends of the type 2 operator site through an interplay of both polar and electropositive residues. Furthermore, we report that CsoR^Sl^ binding drives a ‘conformational switching’ in the operator DNA that is dependent on the inherent deformability brought about by the G/C-tracts within the operator site.

## MATERIALS AND METHODS

### Bioinformatics and macromolecular modelling

DP-bind (27) and DP-dock (28) were used to predict putative DNA binding residues from the amino acid sequence and crystal structure, respectively, of CsoR^Sl^ (11). DP-bind derives a consensus prediction from three machine-learning methods (support vector machine, kernel logistic regression and penalized logistic regression), whereas DP-dock uses a rigid, non-specific and canonical B-DNA probe and ranks predictions based on clustering and interfacial energy. Using both sequence-based and structure-based approaches, a consensus prediction for DNA binding to CsoR^Sl^ was derived. Appropriate protein information (FASTA/PDB) was uploaded to the respective web servers (DP-bind: www.lcg.rit.albany.edu/dp-bind, DP-dock: www.cssb.biology.gatech.edu/skolnick/webservice/DP-dock/index.html), and the programs were run in default mode. For DP-bind results, residues taken from the consensus of support vector machine, kernel logistic regression and penalized logistic regression methods were used. For DP-dock results, a compilation of all candidate residues from clustering and energy-ranked models was used. The 3D-DART web server (www.haddock.science.uu.nl/services/3DDART/) was used to generate 3D models of DNA by providing the appropriate sequence and modelled under default conditions. PDB files of protein and DNA structures were visualized using PyMol (http://www.pymol.org/) or visual molecular dynamics (http://www.ks.uiuc.edu/), and electrostatic maps were generated using CCP4MG (29). Multiple sequence alignments of various biochemically characterized CsoRs were performed using ClustalΩ under default parameters. The UniProt accession codes for the various CsoR entries are as follows: D6EK73 (*S. lividans*), P71543 (*M. tuberculosis*), A6QIT1 (*Staphylococcus aureus*), O32222 (*B. subtilis*), Q8Y646 (*Listeria monocytogenes*) and P64530 for *E. coli* RcnR.

### Site-directed mutagenesis and protein over-expression

Site-directed variants of CsoR^Sl^ (R54A, R57A, Q81A, R129A and R132A) were constructed using a method based on Stratagene’s Quikchange mutagenesis protocol. The forward and reverse mutagenic primers used to introduce the respective mutations are reported in Supplementary Table S1 of Supporting Information. In brief the pET28a plasmid (0.5 ng/μl) containing the full-length CsoR^Sl^ gene was mixed with the desired mutagenic primers (2.5 ng/μl), Pfu Turbo polymerase and buffer (Agilent), dNTPs (0.2 mM) and DMSO (6%), to give a final volume of 30 µl and PCR carried out with the following parameters: 95°C (3 min), [95°C (1 min), 58°C (30 s), 68°C (15 min)] × 15, 72°C (8 min). All mutant clones were sequenced to corroborate that the intended nucleotide changes were successfully introduced. Over-expression of the wild-type CsoR^Sl^ and the mutant proteins was as previously described (11) with masses of all purified proteins determined by denaturing ESI-MS analysis using a Micromass Quattro Ultima triple quadrupole mass spectrometer. Copper-bound CsoR^Sl^ was prepared by introducing an equimolar amount of CuCl (Sigma) to the protein sample in an anaerobic chamber (DW Scientific [O_2_] < 2 ppm). The Cu(I) concentration was determined spectrophotometrically by step-wise addition to a known concentration of the Cu(I)-specific bidentate chelator bicinchoninic acid (BCA) using an extinction coefficient at 562 nm of ε = 7900 M^−^^1 ^cm^−^^1^ for [Cu^I^(BCA)_2_]^3−^ (30).

### Preparation of DNA for binding studies

All DNA was purchased from Sigma and prepared in 10 mM 4-(2-hydroxyethyl)-1-piperazineethanesulfonic acid (HEPES), 150 mM NaCl (pH 7.5) and concentrations of individual oligonucleotides determined using appropriate extinction coefficients at 260 nm on a Nanodrop 2000 (Thermo Scientific). Equal concentrations of complementary strands were annealed by heating at 95°C for 5 min in a water bath and cooled overnight to room temperature. The various CsoR^Sl^ DNA operator sequences used in this work are reported in Table 1.

**Table 1.** The CsoR^Sl^ DNA operator sequences and variants used in this study

DNA name	CsoR^Sl^ operator sequences and variations
*csoR*-CON and *csoR*-EXT	5**′**-CGGACAA**ATACCCCTGGTGG****GTA****TAT**ATGG-3**′**
	3**′**-GCCTGTT**T****ATG****GGGACCACCCATATA**TACC-5**′**
*csoR*-half site	5**′**-CGGACAA**ATACCCCTGG**-3**′**
	3**′**-GCCTGTT**TATGGGGACC**-5**′**
*csoR*-G swap	5**′**-CGGACAAATA**G**CCCTGGTGG**C**TATATATGG-3**′**
	3**′**-GCCTGTTTAT**C**GGGACCACC**G**ATATATACC-5**′**
*csoR*-T swap	5**′**-CGGACAAAT**T**CCCCTGGTGGG**A**ATATATGG-3**′**
	3**′**-GCCTGTTTA**A**GGGGACCACCC**T**TATATACC-5**′**
*csoR*-A swap	5**′**-CGGACAAA**A**ACCCCTGGTGGGT**T**TATATGG-3**′**
	3**′**-GCCTGTTT**T**TGGGGACCACCCA**A**ATATACC-5**′**
*copZA2-*CON and *copZA2-*EXT	5**′**-GCCTT**TATACCCCCTAGGG****GTA**AGGTGGG-3**′**
	3**′**-CGGAA**AT****ATG****GGGGATCCCCAT**TCCACCC-5**′**
*copZA1-*CON and *copZA1-*EXT	5**′**-CGTTGGG**TA****CCCCCTAGGG****GTA****T**ACATGG-3**′**
	3**′**-GCAACCC**ATG****GGGGATCCCCAT****A**TGTACC-5**′**
*copZA1*-GTA up	5**′**-CGTTGG**A**TACCCCCTAGGGGTATACATGG-3**′**
	3**′**-GCAACC**T**ATGGGGGATCCCCATATGTACC-5**′**
*copZA1*-GTA down	5**′**-CGTTGGGTACCCCCTAGGGGTATA**T**ATGG-3**′**
	3**′**-GCAACCCATGGGGGATCCCCATAT**A**TACC-5**′**

*csoR-(4375)*, *copZA1* and *copZA2* are the three DNA operator sites identified to be under the control of CsoR^Sl^ in *S. lividans* 1326. CON refers to a duplex consisting of only the respective consensus operator sequence and is indicated in bold. EXT refers to a duplex that includes the consensus sequence (bold) plus additional flanking genomic nucleotides either side. The GTA motifs discussed in the main text are underlined and changes to the native sequences are highlighted in bold and underlined.

### Ultraviolet-visible and circular dichroism spectroscopy

Protein concentrations were determined by absorption spectroscopy on a Varian Cary 50 ultraviolet (UV)-visible spectrophotometer using an extinction coefficient (ε) of 3105 M^−^^1 ^cm^−^^1^ at 280 nm for the CsoR^Sl^ monomer (11). Far-UV circular dichroism (CD) spectra of protein and DNA samples were measured using an Applied Photophysics Chirascan CD spectrophotometer (Leatherhead, UK) thermostatted at 20°C. Purified protein samples (20 µM) were prepared in 10 mM potassium phosphate and 50 mM potassium fluoride (pH 7.0) and CD spectra measured between 260 and 190 nm. Fraction helicity (*f*_H_) was calculated as *f*_H_ = (θ_222_ − θ_χ_)/(θ_222max_ − θ_χ_), where θ_222_ is the molar residue helicity (MRE) at 222 nm, θ_χ_ is a constant given by [2220 – (53*T)] where T is temperature (°C), and θ_222max_ is the theoretical maximum for 100% helicity given by (−44 000 + 250*T)*(1-*k*/*Nr*) where k is a wavelength constant (*k* = 2.4 at 222 nm) and *Nr* is the number of residues (31,32). For CD scans of DNA and the CsoR^Sl^–DNA complexes, samples were prepared in 10 mM HEPES, 150 mM NaCl (pH 7.5). The A-DNA state of *csoR*-EXT was induced under conditions of 80% v/v trifluoroethanol (TFE), 1 mM HEPES, 15 mM NaCl, 0.3 mM EDTA (pH 7.5). Spectra were measured between 300 and 200 nm using 10 µM of DNA, and analysis of the protein–DNA complex spectrum was carried out after subtracting signals from the free protein in the respective complexes.

### Isothermal titration calorimetry

All calorimetric titration experiments were carried out at 25 ± 0.1°C on a MicroCal VP-ITC calorimeter in 10 mM HEPES, 150 mM NaCl (pH 7.5). Before each run, samples were degassed for 15 min at 23 ± 0.1°C using the ThermoVac accessory. The desired DNA duplex (100 μM) was loaded into the injection syringe and titrated into 10 μM of wild-type or mutant CsoR^Sl^ tetramer present in the sample cell with stirring at 307 rpm for the duration of the experiment. A reference power of 5 μcal/s was used with an initial 3 µl of injection of DNA followed by 6 µl for all subsequent titrations points, a 60 s initial equilibrium delay and 270 s pause between injections. Raw data were analysed using Origin 7.0 software. The integrated data were corrected for the heat of dilution of DNA into buffer, buffer into protein and buffer into buffer, and the binding isotherms were fitted using binding models provided in the software package of the manufacturer. All isothermal titration calorimetry (ITC) experiments reported were carried out in duplicate.

### Size-exclusion chromatography

A Superdex S200 chromatography column (GE-Healthcare) equilibrated in 10 mM HEPES, 150 mM NaCl (pH 7.5), was used to assess the speciation of the CsoR^Sl^:DNA complex after ITC measurements. ITC products were injected onto the column, and the DNA elution profile was monitored at 254 nm.

## RESULTS

### Thermodynamics of CsoR^Sl^ binding to its *csoR* operator

**Figure 2.**
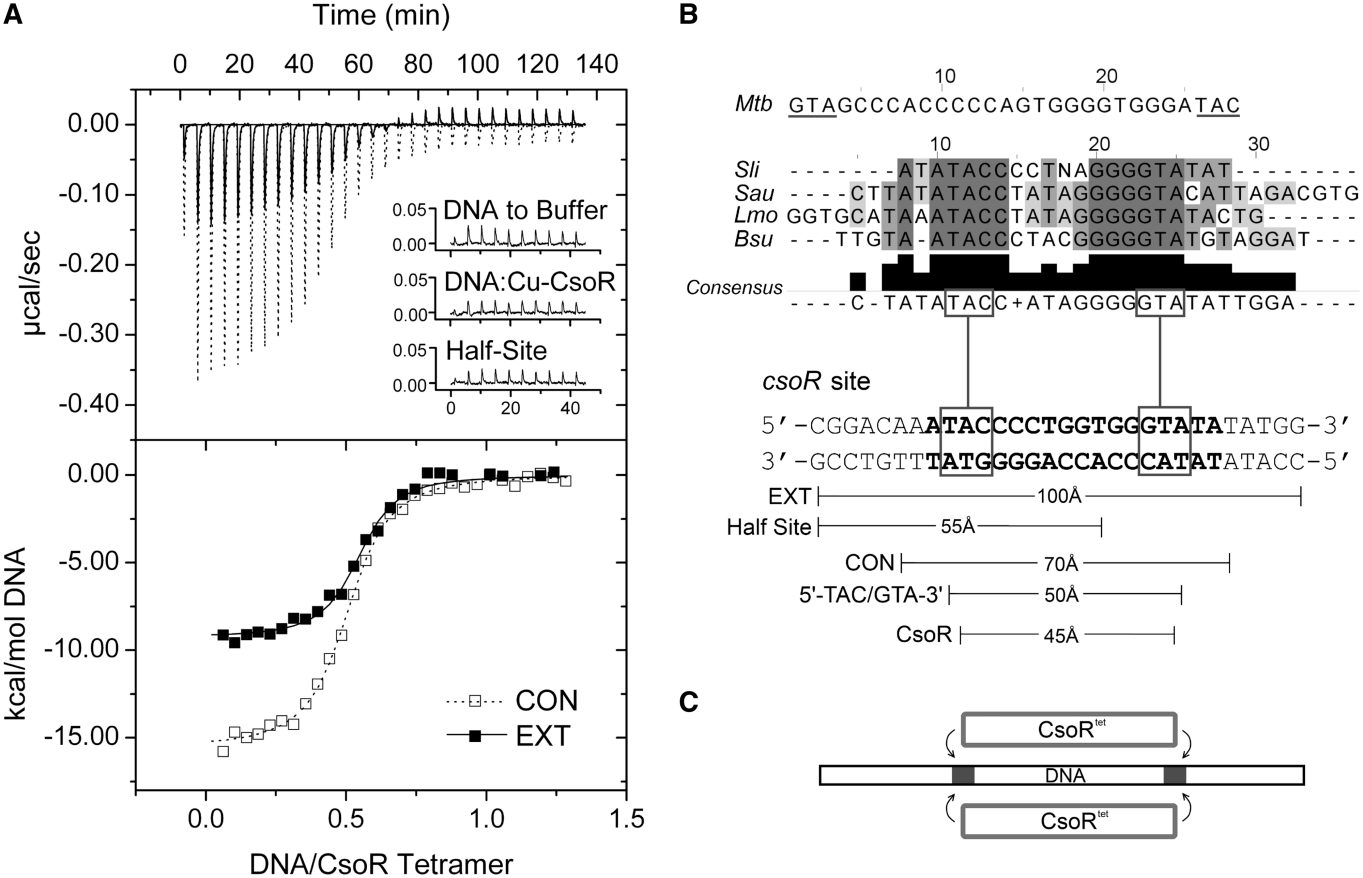
Thermodynamics and operator site symmetry. (**A**) ITC binding profiles at 25°C and fits to a one set of sites binding model (dashed and solid line) for wild-type CsoR^Sl^ with the consensus (CON) and extended (EXT) *csoR* operator (Table 1). A null response similar to DNA dilutions (inset) is observed with Cu(I)-CsoR, as well as with a construct comprising half the *csoR*-EXT sequence (Table 1). (**B**) A sequence alignment of various type 2 CsoR operator sites identified in *S. lividans* [*Sli* (11)], *Staphylococcus aureus* [*Sau* (12)], *Listeria monocytogenes* [*Lmo* (33)] and *B. subtilis* [*Bsu* (8)]. The presence of a 5′-TAC/GTA-3′ inverted repeat is noted as a consistent element in the alignment consensus, with the *M. tuberculosis* binding site having an inverse orientation of this inverted repeat. The approximate lengths of various DNA constructs of *csoR-*EXT used as probes to study the binding characteristics of CsoR^Sl^ are indicated, highlighting similar dimensions to the tetrameric CsoR^Sl^ width with that of the 5′-TAC/GTA-3′ inverted repeat. (**C**) A proposed schematic of the CsoR symmetry on binding to its operator, with the region of the GTA dyads shown as grey regions on the DNA.

We have previously identified a type 2 consensus binding sequence for streptomycetes CsoR orthologues that corresponds to a 21 nt pseudopalindromic sequence ATATACCCCTNAGGGGTATAT where positions 3–8 and 14–19 (underlined) are the most conserved either side of a non-conserved spacer of 5 nucleotides (N5) (11). In *S. lividans* 1326, three consensus-like operator sequences have been identified and shown to bind to CsoR^Sl^ (11). Two of these sites are located upstream of *copZA*-like operons (gene numbers *SLI1317/1318* and *SLI3079/3080*, designated *copZA1* and *copZA2*, respectively; Table 1), which encode for a Cu efflux system consisting of a CopZ-like Cu chaperone (*copZ*) and a P1-type ATPase (*copA*). The third site is located upstream of the CsoR^Sl^ gene, *csoR-4375* (11). These operator sites vary in length with the shortest, *copZA1*, consisting of 16 base pairs, and *copZA2* and *csoR-4375* having 17 and 19 base pairs, respectively (Table 1). Using the operator site with the longest consensus sequence, *csoR-4375* (*csoR*-CON, Table 1), the thermodynamics of binding to apo-CsoR^Sl^ were first investigated by ITC. An exothermic binding isotherm was obtained that could be fit to a single site binding model to give a stoichiometry of binding (*N*) of two CsoR^Sl^ tetramers to one DNA (Figure 2A), consistent with a previous study using analytical gel filtration chromatography (11). The thermodynamic binding parameters are reported in Table 2, where it can be seen that the reaction is highly enthalpically (ΔH_b_) driven with a small unfavourable entropic contribution (-TΔS_b_), leading to an overall favourable free energy of binding (ΔG_b_). The dissociation constant (*K_D_*) is determined to be 120 nM (Table 1). Repeating the experiment with Cu(I)-CsoR^Sl^ did not result in the detection of an exothermic binding isotherm, but instead a small endothermic heat release was observed similar in magnitude to that seen from titrating DNA into buffer (inset Figure 2A and Supplementary Figure S1A and B). This indicates that binding is abrogated when Cu(I) is bound to the CsoR^Sl^ and is in agreement with the mechanism of Cu-induced negative regulation as previously reported for CsoR members (7,8,11).

**Table 2.** Thermodynamic parameters and the stoichiometry of binding (*N*) of the *csoR* operator site binding to wild-type (WT) CsoR^Sl^ and mutants obtained from ITC

DNA	Protein	*N*	*K*_D_ (nM)	ΔG_b_ (kcal mol^−1^)	ΔH_b_ (kcal mol^−1^)	-TΔS_b_ (kcal mol^−1^)
*csoR*-CON	WT	0.54 (0.06)	120 (9)	−9.4 (0.5)	−13.8 (0.7)	4.4 (0.7)
*csoR*-EXT	WT	0.56 (0.05)	74 (9)	−9.7 (1.4)	−11.7 (1.7)	2.0 (0.3)
*csoR*-EXT	R54A	0.24 (0.1)	2062 (667)	−7.8 (3.9)	−5.4 (2.7)	−2.4 (0.2)
*csoR*-EXT	R129A	0.75 (0.02)	134 (13)	−9.4 (0.1)	−17.4 (1.2)	8.1 (1.2)
*csoR*-EXT	R132A	0.53 (0.1)	309 (58)	−8.9 (1.2)	−11.1 (1.5)	2.1 (0.2)

The uncertainties are given in parenthesis and are the standard deviation determined from duplicate measurements.

Experiments were performed at 25°C and pH 7.5. The R57A and Q81A mutants did not give an ITC profile, and therefore no parameters are reported.

### DNA contact of each CsoR^Sl^ tetramer spans the 5′-TAC/GTA-3′ inverted repeats within the pseudopalindromic consensus sequence

To understand how apo-CsoR^Sl^ is localized at its type 2 operator site, the dimensional characteristics of the site were investigated. The base pair length for all operator sites in *S. lividans* correspond to approximately two helical turns of a canonical B-DNA duplex (∼70 Å in *csoR*-CON, Figure 2B), whereas the width of the tetrameric face of apo-CsoR^Sl^ is ∼45 Å (Figure 1B). By adding flanking genomic regions to *csoR*-CON, *csoR*-EXT was created, which is ∼100 Å in length (Figure 2B, Table 1). ITC data using *csoR*-EXT are again consistent with a 2:1 CsoR^Sl^:DNA ratio with a *K*_D_ of 74 nM indicating a small ∼1.5-fold increase in binding affinity compared with *csoR*-CON (Figure 2A, Table 2). The ΔG_b_ is relatively unaffected compared with *csoR*-CON with a more favourable -TΔS_b_ compensating for an observed decrease in ΔH_b_ (Table 2). Notably, the decrease in *K_D_* indicates that additional bases either side of the consensus sequence serve to stabilize the binding interaction of the two CsoR^Sl^ tetramers, or perhaps that operator contact is divided among two tetramers and is stabilized by distal sites external to the consensus sequence. To test the latter, a 17 base pair (∼55 Å, Figure 2B) ‘half-site’ *csoR* construct was designed, consisting of half the *csoR-*EXT sequence and four residues of the N5 region (*csoR*-half site, Table 1). Thus one ‘consensus’ and one ‘distal’ element are preserved. ITC data clearly indicate that the *csoR*-half site results in no detectable binding by apo-CsoR^Sl^ (Figure 2A, and Supplementary Figure S1C) and is therefore consistent with mass spectrometry data (18), providing further evidence that the contact by both CsoR^Sl^ tetramers is contained within the type 2 consensus sequence.

To probe further where CsoR^Sl^ contact is restricted at the consensus operator site, we looked for a common motif across known CsoR operator sites (Figure 2B). A highly conserved 5′-TAC/GTA-3′ inverted repeat flanking G-tracts of variable lengths and spanning 9 base pairs (5′-TACX_9_GTA-3′) is observed among a number of CsoR operator sites (Figure 2B). In the case of CsoR^Mtb^, up to 22 base pairs span this motif, which in contrast is inverted to 5′-GTA/TAC-3′ (7) (Figure 2B). Because the length spanning this inverted repeat (∼50 Å, Figure 2B) is similar to the CsoR^Sl^ tetrameric width of ∼45 Å, CsoR^Sl^ may bind around this region of the consensus sequence. To test whether protein contact is strongly restricted to this motif/region of the DNA, we created constructs of the *csoR-*EXT operator site wherein each nucleotide in the 5′-TAC/GTA-3′ motif has been swapped with its complementary base, and at both ends of the consensus sequence (G/T/A-swap, Table 1). These mutations retain the base composition of the *csoR*-EXT site, but the absence of a binding isotherm and a heat release similar in magnitude to DNA dilutions is a strong indication that CsoR^Sl^ requires this inverted repeat to bind (Supplementary Figure S1D–F). The clear dependence of CsoR^Sl^ on this motif for binding and the absence of binding in the half-site construct therefore suggests an end-to-end contact of CsoR^Sl^ tetramers across the operator site, as illustrated by the cartoon in Figure 2C.

### The formation and binding affinity of the CsoR^Sl^-DNA complex is dependent on a conserved helix α1 -α2 motif and modulated by helix α3 residues

**Figure 3.**
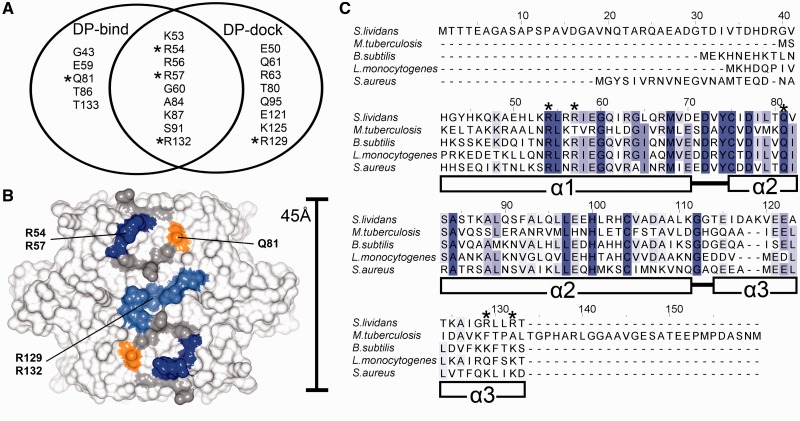
DNA binding predictions and CsoR sequence alignments. (**A**) Venn diagram depicting the CsoR^Sl^ residues predicted from DP-bind and DP-dock to be involved in DNA binding. Chosen residues for mutational studies are marked with asterisks, and their location and conservation on the CsoR^Sl^ tetrameric assembly are highlighted in (**B**) and (**C**), respectively. The indicated secondary structure in (C) is relative to the *S. lividans* CsoR. The location of residues corresponding to the consensus prediction between both DP-dock and DP-bind that were not mutated in this study are coloured grey in (B).

**Figure 4.**
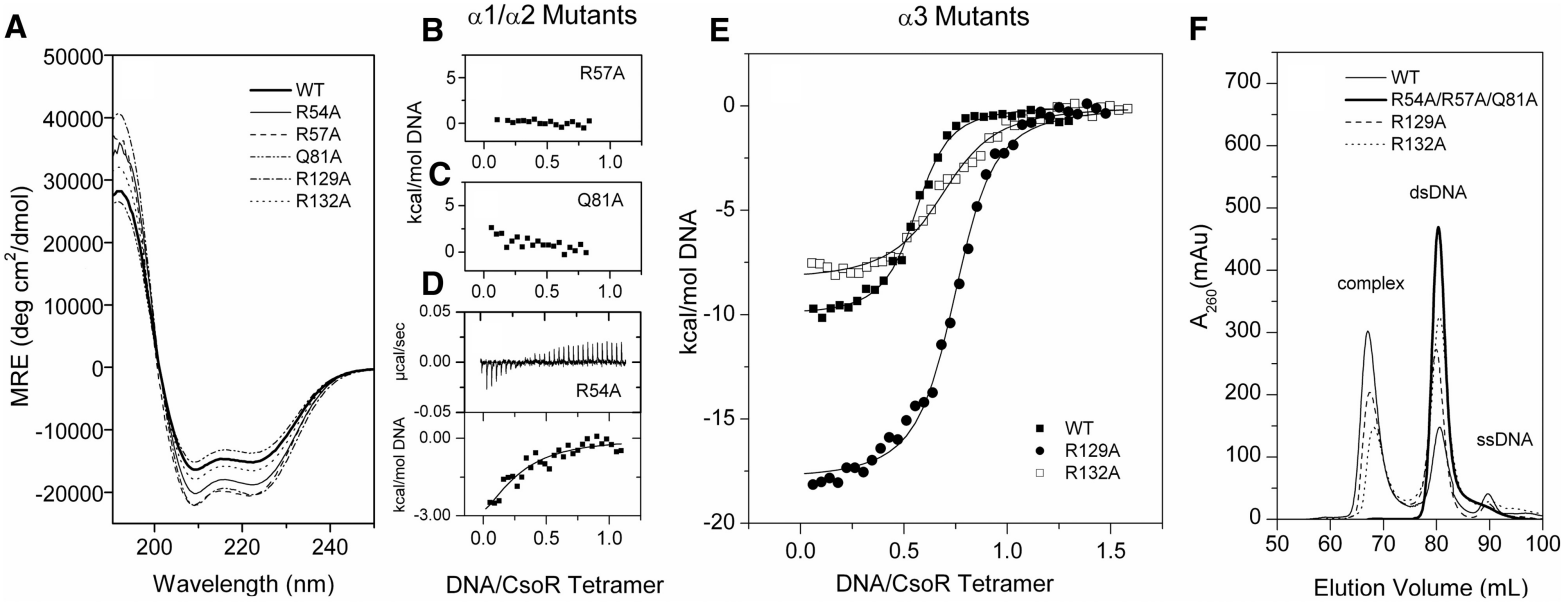
Effects of Ala mutations on DNA binding. (**A**) Far-UV CD profiles of Ala mutants at pH 7.5 showing no considerable conformational differences from the wild-type CsoR^Sl^. (**B–E**) ITC data at 25°C and fit to a one set of sites binding model (solid line) of CsoR^Sl^ Ala mutants on binding to *csoR*-EXT. (B, C, D) Helix α1 and α2 mutants show the most dramatic reduction in affinity, with R54A showing weak binding. (E) Mutations of helix α3 Arg residues whereby R132A changes the binding affinity, and R129A changes the apparent CsoR^Sl^:DNA stoichiometry. (**F**) Gel-filtration elution profiles of the ITC products reveal the absence of a complex peak eluting at 68 ml for the helix α1 and α2 mutations, whereas the helix α3 mutations appear to retain the bound 2:1 complex with DNA.

A combination of machine learning (DP-bind) and rigid docking (DP-dock) algorithms were used to derive *ab initio* inferences regarding the DNA binding residues of CsoR^Sl^ (27,28). The Venn diagram in Figure 3A illustrates a strong consensus prediction between the two programs, where the location of predicted residues appears to be along the electropositive area of the tetrameric face (Figure 3B). From this consensus, we chose to test three Arg residues, R54 and R57 located on helix α1 and R132 located on helix α3. A sequence alignment of biochemically studied CsoR proteins reveals that R54 and R57 are part of a RLXR motif at the α1 helix, which constitutes the ends of the electropositive tract that runs diagonally across each CsoR-tetrameric face (Figure 3C). Residue 1 and 2 of this motif are highly conserved across species, whereas residue 4, with the notable exception of CsoR^Mtb^, is usually an Arg residue (Figure 3C). In contrast, R132 at the α3 is not conserved; however, Lys residues are often found distributed along helix α3 in other sequences (Figure 3C), which have been shown structurally to contribute to the electropositive tracts (7,10,11). Outside of the predicted consensus residues, two other residues were chosen: R129 that is solvent exposed and adjacent to R132 at the end of helix α3, and Q81, located on helix α2, which is completely conserved across all known CsoR sequences (Figure 3C). Q81 is proximal to the α1-RLXR motif and links the α1 and α2 helices in CsoR^Sl^ through a H-bond interaction between its Nδ atom and the NH group of Q61 (11). Arg and Gln residues have distinct abilities to form bidentate interactions with nucleotide bases in protein–DNA complexes (34). The location of residues used in the mutational studies is illustrated in Figure 3B. All mutant proteins eluted from an analytical gel filtration column with a retention volume consistent with a tetramer assembly (data not shown) and the far-UV CD spectra (Figure 4A) reveal each mutant is correctly folded. All mutations are located in an α-helix, and with the exception of the Q81A mutant, the introduction of an α-helix stabilising Ala residue leads to various increases in the percentage helicity relative to WT; Q81A (38%) < WT (41%) < R132A (45%) < R54A (51%) < R57A = R129A (55%). The slight decrease for Q81A may be attributed to the loss of a stabilising polar side chain interaction with the NH group of Q61 in helix α1.

Using *csoR*-EXT, the effect of each Ala mutation on DNA binding was assessed. ITC titrations with the apo-forms of each mutant was carried out and the results summarized in Figures 4B–E and Table 2. The R57A and Q81A mutants both gave small endothermic heat releases on titration with the DNA that were roughly equivalent to that observed when titrating *csoR-*EXT into buffer (Figure 4B and C and Supplementary Figure S1G and H). No fitting of these data to a binding model was possible, and it was concluded that binding by ITC was no longer detectable for these two mutants. This was corroborated from electrophoretic mobility shift assays, where no retardation of the DNA, indicative of complex formation, was observed (Supplementary Figure S2). Furthermore, analytical gel filtration used to monitor the retention volume of DNA at 260 nm gave no peak at ∼68 ml indicative of a 2:1 CsoR^tet^:DNA complex as previously determined for the wt CsoR^Sl^ (11) (Figure 4F). Instead, a peak at ∼81 ml was observed, consistent with free (unbound) *csoR-*EXT (Figure 4F). Likewise, the R54A mutant does not give a complex peak, or a clear band shift in the electrophoretic mobility shift assay experiment (Supplementary Figure S2), suggesting that the binding affinity for *csoR*-EXT by this mutant is also significantly decreased. This is further confirmed from ITC experiments where initial titrations of DNA into apo-R54A resulted in smaller exothermic heat releases (Figure 4D) compared with the wt apo-CsoR^Sl^ (Figure 2A). Fitting the isotherm to a single-site binding model gave a poor fit with an inconsistent *N* value and the thermodynamic parameters reported in Table 2. The detection by ITC of heats of binding for R54A suggests that R57 and perhaps surprisingly, Q81, provide an overall greater contribution to binding affinity.

The helix α3 mutants, R129A and R132A, show contrasting behaviour compared with the helices α1 and α2 mutants (Figure 4E and Supplementary Figure S1I and J). ITC profiles were obtained for both of these mutants on titrating in *csoR-*EXT. The R132A mutant displays a ∼4-fold decrease in affinity for *csoR*-EXT compared with wt CsoR^Sl^, but retains relatively similar ΔH_b_ and –TΔS values (Table 2). R132 is located at the centre of the tetrameric face on helix α3 (Figure 3B), and these results indicate that it clearly contributes to DNA binding affinity, but to a significantly lesser extent than the helix α1 Arg residues R54 and R57. The lower affinity for the R132A is further reflected in the ratios of the ‘complex’ and ‘dsDNA’ peaks in the gel filtration profile (Figure 4F). For the R129A mutant, a ∼2-fold decrease in affinity for *csoR*-EXT is observed from the ITC data (Table 2). This highlights that R132 has the greater contribution to DNA binding of these two helix α3 residues. It is also noted for the R129A mutant that DNA binding coincides with a ΔH_b_ that is significantly more favourable compared with the wt CsoR^Sl^, but the –TΔS term becomes more unfavourable (Table 2). Also of note is the *N* value of 0.75 (Table 2). Although this indicates that the stoichiometry of the complex is no longer 2:1, gel filtration data are inconsistent with this and shows a clear 2:1 complex (Figure 4F). Because unfavourable entropy can be equated to a loss of conformational freedom, it may be that in the absence of R129, the 2:1 complex whilst still highly favourable exhibits compromised dynamics that do not favour the formation of the ‘wt complex’ resulting in an anomalous *N* value obtained form ITC. Thus, R129 can be perceived as being important for specificity with R132 contributing to affinity.

### CsoR^Sl^ binding increases the A-DNA properties of the operator site

**Figure 5.**
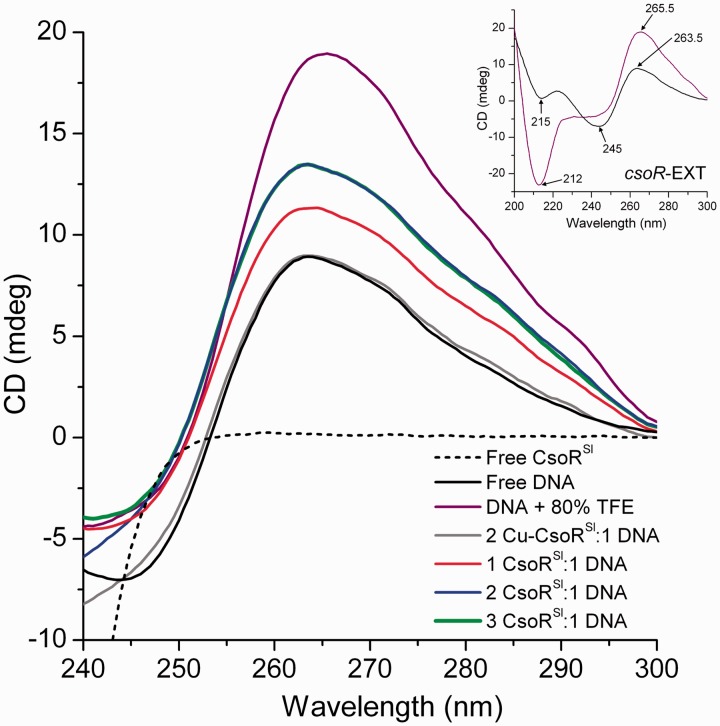
CsoR^Sl^ binding to *csoR*-EXT enhances A-DNA traits. Insert, the CD spectrum of *csoR-*EXT (black) in aqueous solution at pH 7.5 reveals a positive ellipticity at 263.5 nm consistent with A-DNA characteristics ascribed to its G-tract palindromes and retains the same ellipticity in the 260 nm region on induction of true A-DNA form in 80% TFE (purple). No signals from CsoR^Sl^ contribute in the near-UV CD spectrum (black dashed), whereas the ellipticity of *csoR*-EXT (10 µM) in the near-UV region changes in the presence of one (40 µM) and two molar equivalents (80 µM) of CsoR^Sl^ (blue) with no further change in the presence of three molar equivalents (120 µM) of CsoR^Sl^ (green). At two molar equivalents of Cu(I)-CsoR (grey), the near-UV CD spectrum of *csoR*-EXT is the same as in the absence of the apo-CsoR^Sl^. All curves of protein–DNA complexes were baselined with appropriate concentrations of protein and buffer.

The CD spectrum of A-DNA is characterized by positive and negative ellipticity at 260 and 210 nm, respectively, whereas B-DNA is characterized by positive ellipticity at 280 nm, and negative ellipticity at 240 nm (35,36). True A-DNA can only be induced under dehydrating conditions (e.g. with TFE), whereas DNA duplexes of heterogeneous sequence typically exhibit a B-form CD spectrum (35,36). The type 2 operator binding sites of CsoR proteins contain TA-repeats that flank G-tracts of variable lengths (Figure 2B). These duplexes both contribute to positive ellipticity at 260 nm and negative ellipticity at 240 nm, although the contribution at 260 nm for poly-d(G) duplexes is much more dominant (35,36). The high peak intensity at 260 for poly-d(G) duplexes is associated with a distinct A-DNA propensity that imparts a B/A-intermediate conformation in solution (37–39). In agreement with such trends, the CD spectrum of *csoR-*EXT in aqueous solution exhibits a distinct maximum at 263.5 nm, and minima at 245 nm, and 215 nm (inset Figure 5). The max/min at 263.5/215 nm are strong indicators of A-form character that is attributed to the poly-d(G) tracts, whereas the minimum at 245 nm illustrates that B-DNA character is still retained in such duplexes (*csoR-*EXT). In 80%, TFE *csoR*-EXT effectively attains a ‘true’ A-DNA conformation as indicated by the increase in positive ellipiticity at the 260 nm region (peak maxima at 265.5 nm) as well as the appearance of a strong, dominant, negative ellipticity at the 210 nm region (peak minima at 212 nm) that is signature of A-DNA (inset Figure 5). As protein signals do not typically persist in the near UV region (250–300 nm), the effect of protein binding on DNA conformational changes may be observed with out interference from the protein. Thus, we monitored the increase in the 263.5 nm maximum at different ratios of CsoR^Sl^:DNA to investigate the effect of CsoR^Sl^ on the A-DNA signature of the operator site. In aqueous solution, the presence of 1 molar equivalent of CsoR^Sl^ with respect to *csoR*-EXT leads to an increase in the peak maxima at 263.5 nm (Figure 5). At the biologically relevant 2:1 stoichiometry (i.e. 2 molar equivalents of CsoR^Sl^), a further increase in ellipiticity at 263.5 nm is observed, providing strong evidence for a higher A-DNA like character of the operator site on CsoR^Sl^ binding (Figure 5). Beyond a 2:1 stoichiometry, no further increases in the peak at 263.5 nm is observed (Figure 5). Dimensional differences between A- and B-DNA implies that the increase in A-DNA character would have the effect of reducing the distance between the 5′-TAC/GTA-3′ motifs to an average length of ∼41 Å (B-DNA: ∼50 Å, A-DNA: ∼37 Å) and may provide a topology that is more conducive to accommodate the tetrameric CsoR^Sl^ width. Finally, the addition of Cu^I^-CsoR to *csoR*-EXT did not give an increase in ellipticity at 263.5 nm (Figure 5) consistent with the absence of binding as inferred from ITC.

### CsoR^Sl^ binding to the *copZA1* operator is regulated by an external 5′-GTA/TAC-3′ inverted repeat

**Figure 6.**
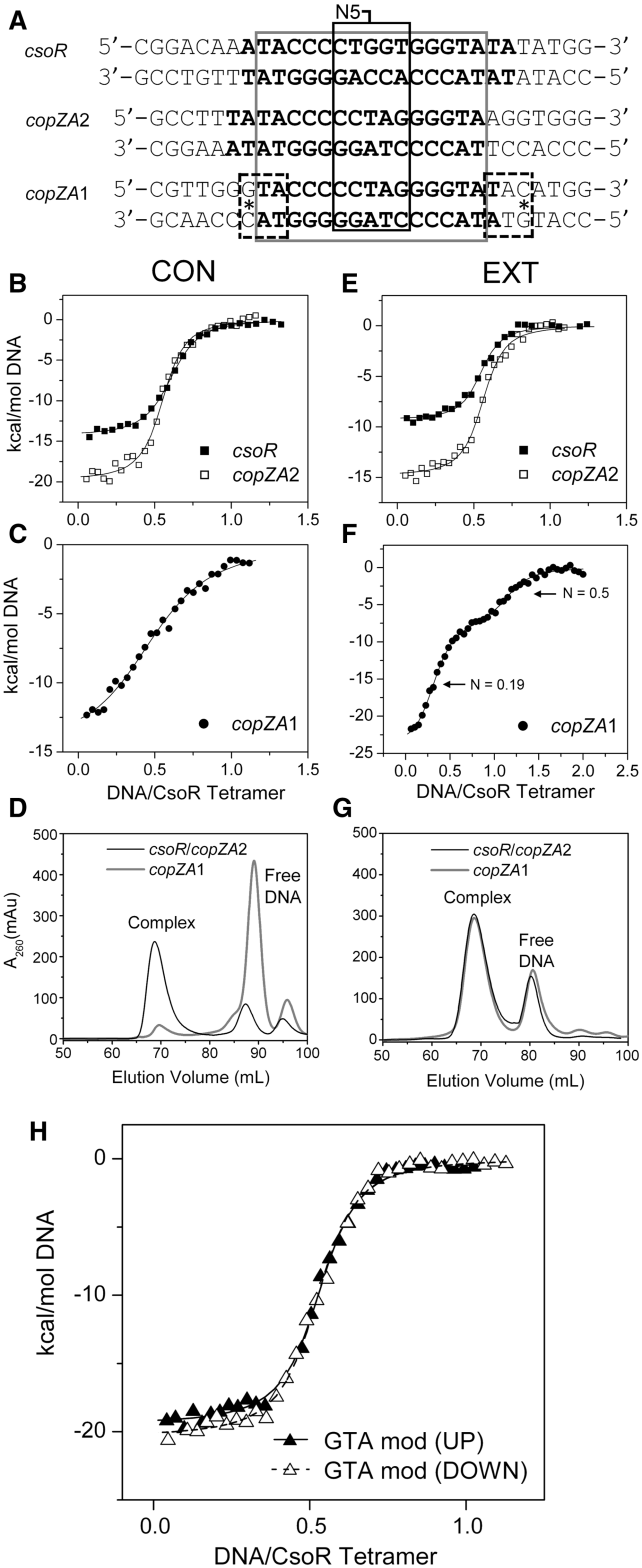
Thermodynamic and gel filtration profiles of the three operator sites regulated by CsoR^Sl^. (**A**) Sequences (left to right: upstream to downstream arrangement) of the three extended (EXT) operator sites for CsoR^Sl^ identified in *S. lividans* 1326 with the consensus sites (CON) indicated in bold, the sequence spanning the 5′-TAC/GTA-3′ inverted repeats boxed in grey, and the variable N5 region indicated. (**B** and **E**) Comparison of binding isotherms obtained from ITC experiments at 25°C with fits to one set of sites binding model (solid line) for wild-type CsoR^Sl^ with *csoR* and *copZA2* operator sites. (**C** and **F**) Binding isotherms obtained from ITC experiments at 25°C for wild-type CsoR^Sl^ with *copZA1*-CON and *copZA1*-EXT. For *copZA1*-CON, the data are fit to a one set of sites binding model (solid line), whereas for *copZA1*-EXT, the data are fit to a two set of sites binding model (solid line) that are saturated at the *N* values indicated. (**D** and **G**) Gel-filtration elution profiles of the ITC products indicating that for *csoR* and *copZA2* -CON and -EXT, a complex peak dominates the profile, whereas for *copZA1*-CON, the weaker affinity determined from the ITC data is corroborated by the free DNA peak dominating the profile. For *copZA1*-EXT a complex peak is observed at a similar elution volume to the 2:1 CsoR:DNA complex. (**H**) Binding isotherms obtained from ITC experiments at 25°C for the G→A mutation of the guanines of the external GTA motifs of the *copZA1*-EXT sequence [dashed boxes and asterisk in (A)] with CsoR^Sl^. Data were fitted to a single site binding model (lines). Representative thermograms obtained from ITC experiments for each construct is shown in Supplementary Figure S3.

Notable differences in the N5 and flanking regions are apparent between all three CsoR^Sl^ operator sites, and this allows for the effect of nucleotide variations on the thermodynamics and mechanism of CsoR^Sl^ binding to be further studied (Figure 6A). Table 3 reports the thermodynamic parameters obtained from ITC data of titrating the *copZA*-CON and *copZA*-EXT into CsoR^Sl^. The stoichiometry of binding remains 2:1 CsoR:DNA for both *copZA1*-CON and *copZA2*-CON, but strikingly the affinity of CsoR^Sl^ for *copZA1*-CON is some 8-fold lower compared with *csoR*-CON or *copZA2*-CON (Table 3, Figures 6B and C). This decrease in affinity for *copZA1*-CON is also implied from gel filtration profiles where the intensity of the free dsDNA predominates over the complex peak at ∼90 ml observed for *copZA2*-CON (Figure 6D). It is notable that both *copZA*-CON sites have a more favourable ΔH_b_ term and a less favourable –TΔS term compared with *csoR*-CON (Table 3), with the latter indicating increased conformational restrictions in the complex. However, for *copZA2*-CON the –TΔS term compensates to yield a ΔG_b_ similar to *csoR*-CON, whereas for *copZA1*-CON the ΔG_b_ becomes marginally less favoured (Table 3). The increases in ΔH_b_ for both *copZA*-CON sites suggests more interactions occur in these complexes, which possibly arise due to variations within the N5 region compared with *csoR*-CON (Figure 6A).

**Table 3.** Comparison of the thermodynamic parameters and stoichiometry of binding (*N*) of the three operator sites binding to wild-type CsoR^Sl^ obtained from ITC

DNA operator	*N*	*K*_D_ (nM)	ΔG_b_ (kcal mol^−1^)	ΔH_b_ (kcal mol^−1^)	-TΔS_b_ (kcal mol^−1^)
*csoR*-CON	0.54 (0.06)	120 (9)	−9.4 (0.5)	−13.8 (0.7)	4.4 (0.7)
*csoR*-EXT	0.56 (0.05)	74 (9)	−9.7 (1.4)	−11.7 (1.7)	2.0 (0.3)
*copZA2*-CON	0.52 (0.04)	107 (12)	−9.5 (1.2)	−18.1 (2.3)	8.7 (2.2)
*copZA2*-EXT	0.53 (0.03)	57 (10)	−9.9 (0.6)	−13.5 (0.7)	3.6 (1.0)
*copZA1*-CON	0.55 (0.01)	971 (86)	−8.2 (0.7)	−16.1 (1.4)	7.9 (1.5)
*copZA1*-EXT	0.19 (0.03)^a^	11 (3)^a^	−10.9 (1.6)^a^	−32.0 (4.8)^a^	21.1 (4.9)^a^
	0.56 (0.03)^b^	162 (21)^b^	−9.3 (1.6)^b^	−9.1 (1.5)^b^	−0.1 (1.5)^b^
*copZA1*-GTA up	0.54 (0.02)	96 (10)	−9.6 (0.08)	−19.6 (0.2)	10.0 (0.2)
*copZA1*-GTA down	0.53 (0.02)	87 (14)	−9.6 (0.7)	−21.5 (1.6)	11.9 (1.6)

^a,b^Parameters obtained from fitting the data to a two sets of sites site binding model, i.e. ^a^*N*_1_ and ^b^*N*_2_, ^a^*K*_D1_ and ^b^*K*_D2_ and so forth. The uncertainties are given in parenthesis and are the standard deviations determined from duplicate measurements.

Experiments were performed at 25°C and pH 7.5.

For the *copZA2*-EXT operator sequence, the ITC profile is similar to *csoR*-EXT with little change in *K*_D_ and a similar trend in ΔH_b_ and –TΔS as noted with *copZA2*-CON is observed (Table 3, Figure 6E). In contrast, the binding isotherm of *copZA1*-EXT is indicative of two binding sites with different affinities, which when fitted to a binding model for 2 sets of sites (Figure 6F) results in the thermodynamic parameters reported in Table 3. Titration of single stranded *copZA1*-EXT showed no response to CsoR^Sl^, ruling out the possibility of interactions with erroneous or self-annealed duplexes. From the isotherm shown in Figure 6F, the first inflexion point corresponds to a high affinity ‘site/species’ and has an *N* value of 0.19, suggesting a CsoR:DNA ratio of 5:1, whereas a second *N* value of 0.56 corresponds to a CsoR:DNA of 2:1 and a *K*_D_ of 162 nM (Table 3). From the gel filtration profile in Figure 6G, the complex elutes at an elution volume identical to *csoR*-EXT and *copZA2*-EXT with the ratio of the complex and free DNA peaks identical in all three cases. Attempts to reconstitute a 5:1 and also a 10:1 CsoR:DNA complex do not alter the 2:1 ratio peak at 68 ml in the gel-filtration profile (data not shown). Equally anomalous is a reverse titration (100 µM CsoR tetramer into 10 µM *copZA1* DNA) giving binding parameters with an *N* (CsoR:DNA) = 0.86 and a reduced enthalpy of binding compared with the forward (DNA into Protein) titration (Supplementary Figure S4). An inspection of the sequence flanking the *copZA*1 consensus reveals an external 5′-GTA/TAC-3′ motif, which overlaps the terminal base pairs of the consensus (dashed boxes Figure 6A). Knowing that such a motif present in the consensus sequence strongly dictates the binding of CsoR^Sl^ (*vide supra*), constructs in which the upstream or downstream guanines of the external 5′-GTA/TAC-3′ motif in *copZA1*-EXT were changed for adenine were designed (*copZA1*-GTA up and *copZA1*-GTA down, respectively, Table 1). Titrations of these duplexes into CsoR^Sl^ gave ITC profiles akin to *csoR*-EXT and *copZA2*-EXT (Figure 6E) where the initial tight binding phase is absent, and the *N* and *K*_D_ values are now aligned with the other -EXT duplexes (Table 3, Figure 6H). The change to an adenine also has the effect of making the binding more enthalpically favourable, with the –TΔS term contributing unfavourably to the ΔG_b_ (Table 3).

## DISCUSSION

### The thermodynamic signatures of CsoR^Sl^-DNA binding suggest a binding mode that involves binding to the major grooves of a linear non-bent operator DNA

From a thermodynamic perspective, the binding of CsoR^Sl^ to all three of its type 2 consensus operator sites is strongly enthalpically driven (|ΔH| > |-TΔS|) (Table 3), which is reminiscent of major groove binding, or phosphate contacts (40). Minor groove binding on the other hand is usually entropically driven (|ΔH| < |-TΔS|). This is largely a result of the entropically favourable event of displacing the spine of hydration along the minor groove of DNA that is prevalent in poly-d(T/A) duplexes (40). Protein structural motifs such as helix-turn-helix or winged helix typically bind at the major groove (17,41) or comprise of domains that trail along the phosphate backbone (e.g. mTERF) (42). Minor groove binding is typically associated with a pronounced kinking of DNA as seen with the β-domains of the TATA-binding protein (43) and with the binding of protein α-helical elements causing a considerable distortion of the cognate DNA into the A-form by widening the minor groove, which is most exemplified in HMG box proteins (44–47). In such cases, groove-binding helices are relatively short and are bridged by flexible loop regions as opposed to the continuous helices found in CsoR faces (7,11). A comparison of thermodynamic signatures for both CsoR and RcnR reveal that although RcnR appears to bind at 25°C with a favourable binding entropy (−TΔS_b_ = ∼−4 kcal/mol) in contrast to CsoR^Sl^ (−TΔS_b_ = ∼4 kcal/mol), association with the respective operator sequences are in both cases enthalpically driven (19). DNA footprinting experiments with RcnR infers that DNA binding is dominated by minor groove contact at the TACT/AGTA inverted repeats (19). However, this does not appear to be consistent with the thermodynamic signature obtained from ITC data: the favourable entropy of RcnR binding to its operator site is not suggestive of base-specific contacts at the minor groove or rather such an event does not appear to incur a considerable entropic cost (where |ΔH| < |−TΔS|). This then is more likely to reflect interaction with backbone phosphates in the minor groove. For CsoR^Sl^, the unfavourable entropy of binding (Table 3) may be a result of conformational entropy from the restricted flexibility in the event of two CsoR tetramers binding to one DNA as opposed to only one in the case of RcnR. These thermodynamic differences are indicative of divergent mechanisms operative between different types of operator sites in the CsoR/RcnR family.

### High-affinity DNA binding by CsoR extends beyond electropositive contact

Through site-directed mutagenesis of CsoR^Sl^, it is established that binding to the operator site is highly dependent on an RLXR motif positioned within the α1 helix (Figure 3), which is relatively conserved across both predicted and functionally characterized CsoR members. A previous study has shown that a double mutation in CsoR^Mtb^, R15A/R52A, where R15 is the R1 residue in the RLXR motif and R52 is located on the α2 helix but not conserved, abrogates binding to the operator sequence (7). In the present study, removal of either R1 (R54) or R4 (R57) has a dramatic effect on CsoR^Sl^ binding to the operator site (Figure 4). By contrast, the α3 helix Arg mutations (R129A and R132A) give a less pronounced effect on binding affinity (Figure 4 and Table 2), which highlights the dominating contribution of the RLXR motif to the binding. A surprising finding is the strong contribution the conserved Q81 residue of CsoR^Sl^ has on DNA binding. This highlights that in addition to electrostatic protein–DNA interactions, polar contacts are also necessary for strong DNA binding and is corroborated further by the number of polar residues predicted to participate in DNA binding from our DNA binding predictions (Figure 3A). The sensitivity of what are essentially mutations at the outer ends of the tetrameric assembly suggests that each tetramer must facilitate high-affinity contact at two ends of the DNA that are stabilized by residues in between, i.e. the helix α3 Arg residues. Because electropositive contact towards DNA is prevalent among DNA binding domains, we propose that based on the degree of DNA binding abrogation observed in certain protein mutations, the RLXR motif at the α1 helix must be, if not constitute, a recognition helix, which together with the juxtapositioned Q81 constitutes a high-affinity binding region for the CsoR^Sl^ operator site. Residues located in between these regions (R129 and R132) clearly act to stabilize interactions with the intermediate nucleotides. These single protein mutations represent what is essentially a loss of four contacts on DNA, and credence to the role of the RLXR motif and Q81 is established by how the presence of such residues can compensate for a loss of contact from the α3 helix Arg residues.

### GTA dyads in CsoR operator sites are high-affinity ‘anchor points’ of protein contact

The binding to a palindromic operator site is a common occurrence among metalloregulatory proteins (2). In *S. lividans*, the type 2 operator sites identified for CsoR^Sl^ all possess a conserved, symmetrical element consisting of a trinucleotide dyad 5′-TAC/GTA-3′ (‘GTA dyad’) that bridges semi-continuous G-tracts with TA-repeats that flank them (Figure 2B). CsoR^Sl^ binding appears to be highly dependent on the arrangement of nucleotides in this motif, as observed from mutations to these individual nucleotides, which abolish detectable DNA binding by ITC at 25°C. When this symmetry is broken, as seen in the half-site construct (*csoR*-half-site), binding is also abolished and gives credence to the dyad axis of symmetry operative in the CsoR–DNA interaction. This sensitivity towards the GTA dyad is similar to that observed for the AGTA dyad in the binding site of RcnR, illustrating a common determinant for high-affinity protein recognition among the different types of CsoR/RcnR operators. The function (inferred from the extent of binding loss from mutations) as well as symmetry between the RLXR motif of the CsoR^Sl^ protein has a certain degree of correspondence to that of the GTA dyads. Both appear to be required for high-affinity binding, with the distance between the RLXR motifs on each tetrameric face (∼40 Å; R57-Cα→R57′-Cα) able to accommodate the length between the GTA dyads (∼50 Å, TAC→GTA). Such notions are indicative of some degree of correspondence between these elements, which we suggest controls how protein contact is divided among the operator site.

### The topology and deformability of the operator site supports the symmetrical binding of two CsoR tetramers

A further unique element of CsoR binding sites is the palindromic G-tracts between the GTA dyads (Figure 2B). Poly-d(G) tracts are associated with a propensity for the A-DNA conformation, which is the result of favourable stacking interactions between guanine bases that is followed by a decrease in propeller twist (48). The canonical A-form is typically induced in dehydrating conditions and results in a conformation with a deeper narrower major groove and a shallower wider minor groove. In aqueous solution, a combination of A-form propensities as well as the effects of hydration in poly-d(G) duplexes results in a unique and well-characterized conformation that is ‘neither A nor B’ (48,49). Structural characterization of this so-called ‘B/A-intermediate’ structure in poly-d(G) duplexes has suggested that its unique topological characteristics may restrict non-specific protein contact along the DNA grooves—a mechanism by which most transcriptional regulators achieve specificity (48). Poly-d(G) duplexes exhibit a unique CD spectrum, most notably having a dominant positive peak maximum at ∼260 nm that is characteristic of a contribution from guanine-stacking interactions (39). A minimal contribution to positive ellipticity at 260 nm is also ascribed to poly-d(TA) duplexes, although these duplexes are distinctly of the B-form (36). True to this, the near-UV CD spectrum of *csoR-*EXT exhibits a signature A-DNA maxima at 263.5 nm under native conditions (Figure 5). This peak maxima is reminiscent of the 270 nm peak observed for the TACT-G_6_-N-AGTA RcnR operator site for which the topological contribution of these G-tracts in protein recognition has been implied (19). An increase in ellipticity at 263.5 nm is a good indication for an increase in A-DNA character and subsequently is observed on the addition of up to 2 molar equivalents of CsoR^Sl^ (Figure 5). Addition of TFE to *csoR*-EXT results in the formation of A-DNA where the peak maximum at 265.5 nm has a clearly higher intensity than in the presence of CsoR^Sl^ (Figure 5). This serves to highlight that CsoR binding stabilizes an enhanced A-like DNA character at the operator site but does not induce an A-form to the same extent as the addition of TFE (Figure 5). A-form characteristics also attain a more symmetrical shape of the phosphate backbone between strands, providing a conducive topology that complements the equally symmetrical binding orientation of two CsoR^Sl^ tetramers. Indeed, conformational selectivity is much more documented for the B- and B′-DNA forms (50,51), where the A-form operator site between GTA dyads is in contrast to the NikR-DNA complex where the DNA length accommodates the protein and favours a B-DNA conformation due to the A/T-rich operator site, which is strengthened by non-specific interactions (52,53).

We propose that the increase in A-form characteristics on CsoR^Sl^ binding is equated to the re-ordering of the shell of water molecules around the operator site, which can be ascribed to conformational entropy as reflected in the thermodynamics of binding. Indeed if A-form characteristics were a result of minor groove widening through direct readout, this would have resulted in an appropriate thermodynamic signature (i.e. endothermic thermograms and an enthalpically driven reaction). Additionally, both poly-d(G) and poly-d(TA) duplexes are highly deformable (37,48), and this property may be taken advantage of by CsoR^Sl^. True to the assumption previously made in the type 1 tandem operator site of RcnR (19), these A/B-hybrid sites between promoter elements are key factors for a mechanism of CsoR recognition based on conformational selectivity.

### A binding model for CsoR^Sl^

**Figure 7.**
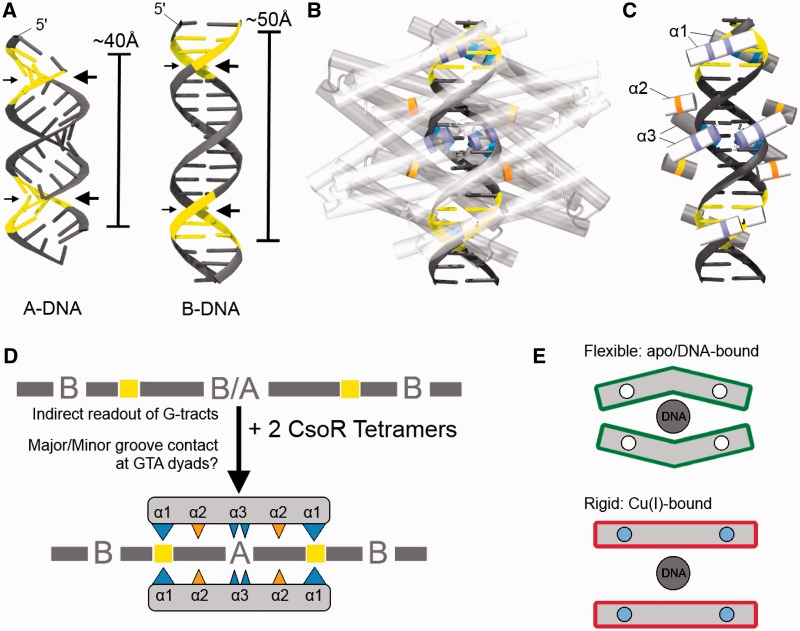
A schematic representation of the CsoR^Sl^–DNA interaction. (**A**) The *csoR*-CON operator site modelled in the A- and B-forms using 3D-DART, showing clear differences in groove orientations and lengths. The major grooves (large arrows) of the GTA dyads (yellow) appear to be syn-facing and suggests that CsoR^Sl^ contact must approach asymmetric orientations on the operator DNA, with that of the second CsoR^Sl^ binding towards the syn-facing minor grooves of the GTA dyads (small arrows). (**B**) The envisaged binding interaction of two CsoR tetramers is shown with B-DNA. Major groove contact is shown in the superimposed tetramer below the DNA (dark), whereas minor groove contact is shown in the tetramer above the DNA (light). (**C**) The proposed interaction involving the residues used in the mutational studies are indicated in their respective helical segments. The blue/purple indicates Arg residues at the helix α1 RLXR motif and the helix α3, and the orange/yellow represents the Q81 at helix α2. (**D**) Cartoon summary of the mechanism proposed for CsoR^Sl^ binding to its operator site. (**E**) Cartoon (transverse view) to illustrate the flexible asymmetric nature of each apo-CsoR^Sl^ tetramer bound to DNA showing how the α-helical movement may occur parallel to the helical length. The proximity of the α1 helices to the Cu-binding site may imply that on binding Cu(I), this flexibility may be lost leading to dissociation of the CsoR. White circles indicate Cu(I)-free, and blue circles indicate Cu(I) bound.

Based on our findings and others, a model describing the CsoR^Sl^–DNA interaction must account for a binding of two CsoR tetramers to the operator DNA that occurs with a 2-fold axis of symmetry, where each tetrameric face contacts one face of the operator. A-DNA and B-DNA models of the *csoR*-EXT site shows that the GTA dyads have *syn*-facing grooves spanning 1.5 turns (Figure 7A). Starting at the GTA dyad sites, each face of the DNA will then have a ‘major face’ consisting of major-minor-major groove arrangements, or a ‘minor face’ with minor-major-minor groove arrangements, and implies that each tetramer would facilitate different contacts on each face (Figure 7A). The results from our study imply that the helix α1 RLXR motif establishes contact at the GTA dyad regions of the type 2 operator site, and this would lead to the positioning of an Arg-rich cluster (RLXR) towards these major or minor grooves of the GTA dyads (Figure 7B). Arg residues are known to have a preference for hydrogen bonding to purines (G/A): guanine at the major groove and adenine at both major and minor grooves (34). These notions suggest that one CsoR^Sl^ tetramer binds with the RLXR motif pointed towards the major face and the second CsoR tetramer binds with the RLXR motif towards the minor face where the functional groups of the purines of the GTA dyads are likely to be exposed. An interplay of major and minor face binding may perhaps drive the high-affinity recognition that is inferred from both protein and DNA mutations. Orientating the CsoR tetramers in this manner conveniently brings the R129, R132 and Q81 residues proximal to the G-tracts between the GTA dyads (Figure 7B and C). Whereas the helix α3 Arg residues are more proximal to the middle groove of the major/minor faces of the DNA, the Q81 appears to be positioned towards the phosphate backbone. We propose that this species conserved Q81 strongly promotes deformability of the G-tracts and in concert with R129 and R132 provides an environment that serves to lock the G-tracts into an A-like conformation. This shape readout of the operator strongly points to a mechanism in which CsoR^Sl^ is also selective to a sequence-based conformational polymorph created by G-tracts between GTA dyads. A-form characteristics essentially attain a more symmetrical topology of the phosphate backbone, an effect that supports the equally symmetrical binding orientation of two CsoR^Sl^ tetramers. From a thermodynamic perspective, it is still unclear whether the binding of two CsoR tetramers occurs in a sequential or simultaneous manner, particularly in terms of driving A-form characteristics at what appears to be two distinct sites (major and minor faces) on a single operator. However, our model suggests that the operator site topology allows both CsoR tetramers to bind similar groove dimensions. CsoR specificity towards type 2 sites amongst species may be due to variable A-form polymorphs reflecting the variability in G-tract length and symmetry amongst such sites (Figure 2B). Differences in G-tract length and continuity between type 1 and type 2 sites likely complement equally distinct sequence variations across CsoR/RcnR proteins, which may account for their respective specificities. For example RcnR has a RASK motif in place of the RLXR motif commonly found in CsoRs (Supplementary Figure S5). A cartoon mechanism to summarize the previously discussed features of CsoR^Sl^ DNA binding is shown in Figure 7D. Finally, the question arises of how Cu-binding drives de-repression of CsoR. As each CsoR tetramer has two identical DNA binding faces, we posit that the ‘relaxed’ apo CsoR flexes towards one face on binding DNA, promoting asymmetry on the opposite face of the tetramer, which may also affect the distance between the Cu(I) ligands. This may account for how only one face of each CsoR tetramer binds DNA, with Cu(I) binding likely restricting this type of movement, and draws the tetramer into a flat, ‘taut’ conformation resulting in complete dissociation on filling all Cu(I) sites (Figure 7E).

### An external GTA dyad has implications in modulating the response to Cu stress

From the three operator targets recognized by CsoR^Sl^, external GTA dyads to the *copZA1* consensus appear to play a role in creating a high-affinity binding site that compensates for the low affinity of the *copZA1*-CON (Figure 6, Table 3). It is clear from the ITC profile in Figure 6F that in the presence of excess CsoR^Sl^ the external GTA dyads in *copZA1* allow a high-affinity site to be occupied first (11 nM), which on saturation leads to the detection of a second site with an affinity and stoichiometry reminiscent of the *copZA2*/*csoR-*CON operator sites (Table 3). The reverse titration also shows an anomaly in the stoichiometry of binding, but the high-affinity site (*N* = 0.19) appears to have been masked (Supplementary Figure S4). Gel-filtration profiles however clearly show that a 2:1 profile persists. Modelling the *copZA1* site as a canonical B-DNA shows that the external GTA dyads create *anti*-facing grooves, which in turn can be responsible for a number of different orientations for CsoR^Sl^ to bind. It may be that initial binding stoichiometry of 5:1 deduced from the forward titration and the anomalous stoichiometry obtained from the reverse titration (Supplementary Figure S4) may relate to some ‘non-specific’ binding mode(s), which on removal of either external GTA dyad abolishes the initial higher affinity binding site (Figure 6H). These observations are perhaps credence to how the presence of external GTA dyads in CsoR operator sites regulates a distinct binding mode compared with that of canonical CsoR^Sl^ operator sites. RNA-seq data of *S. lividans* 1326 have indicated that under Cu homeostasis conditions the *copZ-3079* and *copA-3080* transcripts (*copZA2*) are constitutively expressed, whereas transcripts for *copZ-1317* and *copA-1318* (*copZA1*) are not (11). Under Cu stress the *copZA2* transcript level shows a 5-fold increase whereas *copZA1* remains at a basal level. This was suggested to illustrate the possibility of a modular response to Cu stress, where under homeostasis and/or low cytosolic Cu concentrations, the *copZA2* operon is predominately operative and at higher Cu concentrations *copZA1* then responds (11). Thus, differences observed *in vitro* for the binding between *copZA*2/*csoR* and *copZA*1 operator sites therefore reflects transcriptional differences *in vivo* and is in keeping with a deeper mechanism of Cu-induced regulation of these genes.

## SUPPLEMENTARY DATA

Supplementary Data are available at NAR Online.

## FUNDING

Funds from the University of Essex. Funding for open access charge: Waived by Oxford University Press.

*Conflict of interest statement*. None declared.

## Supplementary Material

Supplementary Data
