# Supplementary material for: Conformational and thermodynamic hallmarks of DNA operator site specificity in the copper sensitive operon repressor from Streptomyces lividans
Source: Nucleic Acids Res. 2013 Oct 8;42(2):1326–40. doi: 10.1093/nar/gkt902 (PMC3902906; doi:10.1093/nar/gkt902)
Supplement: Supplementary Data [file supp_42_2_1326__index.html]

Conformational and thermodynamic hallmarks of DNA operator site specificity in the copper sensitive operon repressor from Streptomyces lividans — Conformational and thermodynamic hallmarks of DNA operator site specificity in the copper sensitive operon repressor from Streptomyces lividans — Supplementary Data 

# Conformational and thermodynamic hallmarks of DNA operator site specificity in the copper sensitive operon repressor from *Streptomyces lividans*

## Supplementary Data

files

**Files in this Data Supplement:**

- Supplementary Data - docx file
