# Supplementary material for: Conformational and thermodynamic hallmarks of DNA operator site specificity in the copper sensitive operon repressor from Streptomyces lividans
Source: Nucleic Acids Res. 2013 Oct 8;42(2):1326–40. doi: 10.1093/nar/gkt902 (PMC3902906; doi:10.1093/nar/gkt902)

**SUPPORTING INFORMATION**

**Conformational and thermodynamic hallmarks of DNA operator site specificity in the copper sensitive operon repressor (CsoR) from *Streptomyces lividans***

Benedict G. Tan^1^, Erik Vijgenboom^2^ and Jonathan A.R. Worrall^1*^

^1^School of Biological Science, University of Essex, Wivenhoe Park, Colchester, CO4 3SQ, UK. ^2^Molecular Biotechnology, Institute of Biology Leiden, Sylvius Laboratory, Leiden University, PO Box 9505, 2300RA Leiden, The Netherlands.

*To whom correspondence should be addressed: email; jworrall@essex.ac.uk Tel; +44 1206 872095

**Table S1:** Mutagenic primer pairs used to generate the various mutations in CsoR^Sl^. In lower case are the nucleotides that are changed from the original sequence to generate the respective CsoR^Sl^ mutation.

| **Primer** | **Length** | **GC**  **%** | **Tm**  **(°C)** |
| --- | --- | --- | --- |
| **R54A**  Forward  5'-GCACCTCAAAgcCCTGCGCCGCATCG-3'  Reverse  5'-CGATGCGGCGCAGGgcTTTGAGGTGC-3' | 26 | 70 | 62 |
| **R57A**  Forward  5'-CAAACGCCTGCGCgcCATCGAGGGCC-3'  Reverse  5'-GGCCCTCGATGgcGCGCAGGCGTTTG-3' | 26 | 73 | 64 |
| **Q81A**  Forward  5’-CGACATACTGACCgcGTCTCCGCCTCCACG-3’  Reverse  5’- CGTGGAGGCGGAGACgcGGTCAGTATGTCG-3’ | 30 | 67 | 65 |
| **R129A**  Forward  5’-GGCCATCGGCgcGCTACTGCGCACG-3’  Reverse  5’-CGTGCGCAGTAGCgcGCCGATGGCC-3’ | 25 | 76 | 64 |
| **R132A**  Forward  5’-GGCAGGCTACTGgcCACGTGAGCGCTC-3’  Reverse  5’-GAGCGCTCACGTGgcCAGTAGCCTGCC-3’ | 27 | 70 | 64 |

**Figure S1:** Raw ITC data for wild type CsoR^Sl^ and various variants titrated with buffer or the *csoR*-EXT operator sequence as well as nucleotide variants within the *csoR*-EXT sequence, as described in the main text. All data collected at 25 ^o^C.


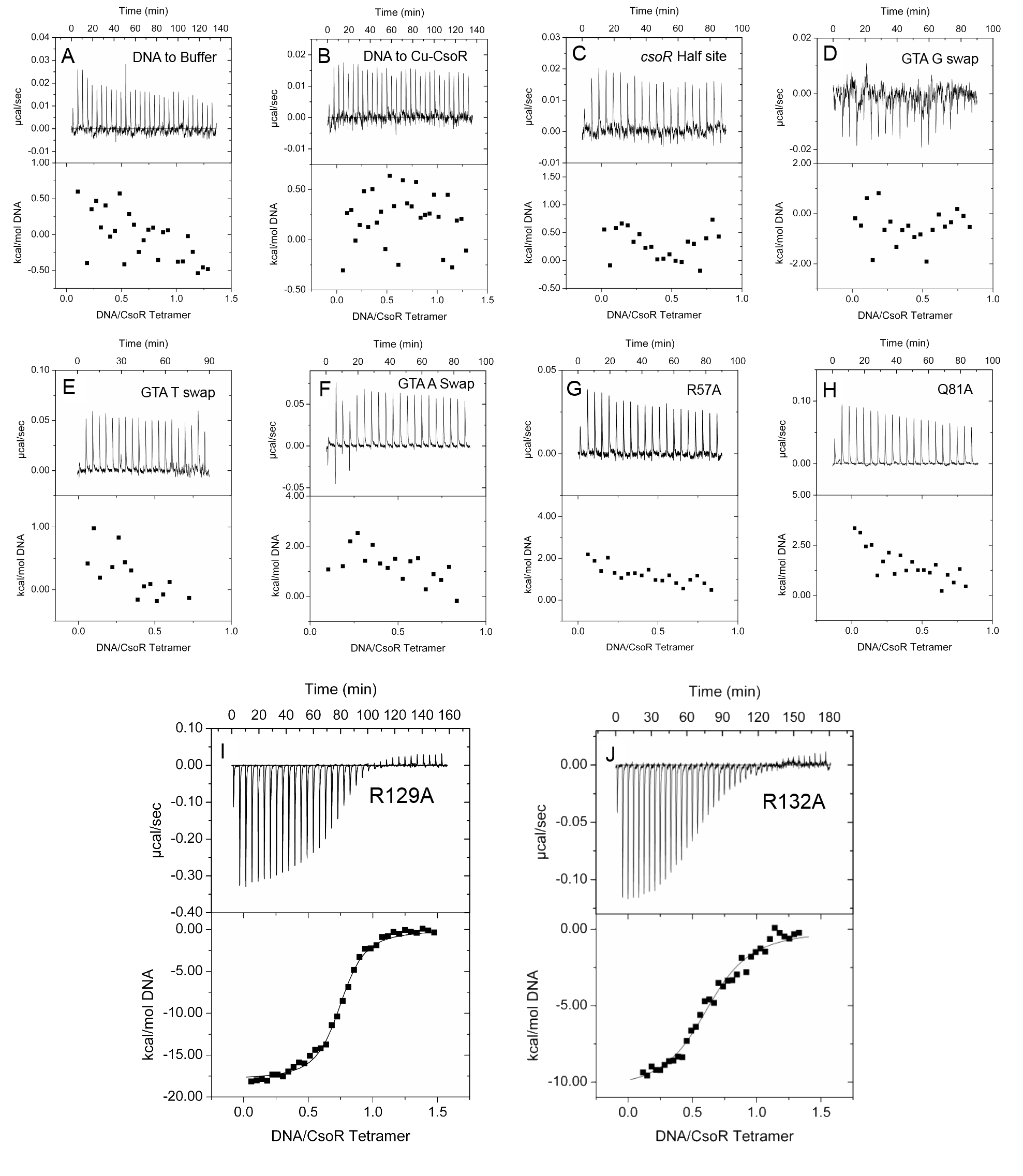


**Figure S2:** Electrophoretic mobility shift assays of wild type CsoR^Sl^ and variants in the presence and absence of Cu(I) using the *csoR*-EXT operator sequence as the binding target. Samples were prepared by incubating 1 μM of *csoR*-EXT with 10 μM apo-CsoR^Sl^ or Cu(I)-CsoR^Sl^ in 10 mM HEPES, 150 mM NaCl, pH 7.5 for 30 min at room temperature, followed by loading to a 6% polyacrylamide gel which was pre-run at 170 V for 1 h in Tris-Borate EDTA (TBE) buffer. Gels were stained with ethidium bromide for 30 min and visualized under UV light.


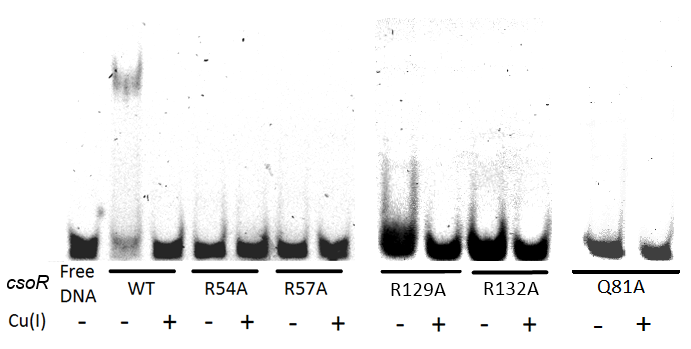


**Figure S3:** Raw ITC data of wild type CsoR^Sl^ titrated with the three operator targets and their nucleotide variations as discussed in the main text. All data collected at 25 ^o^C.


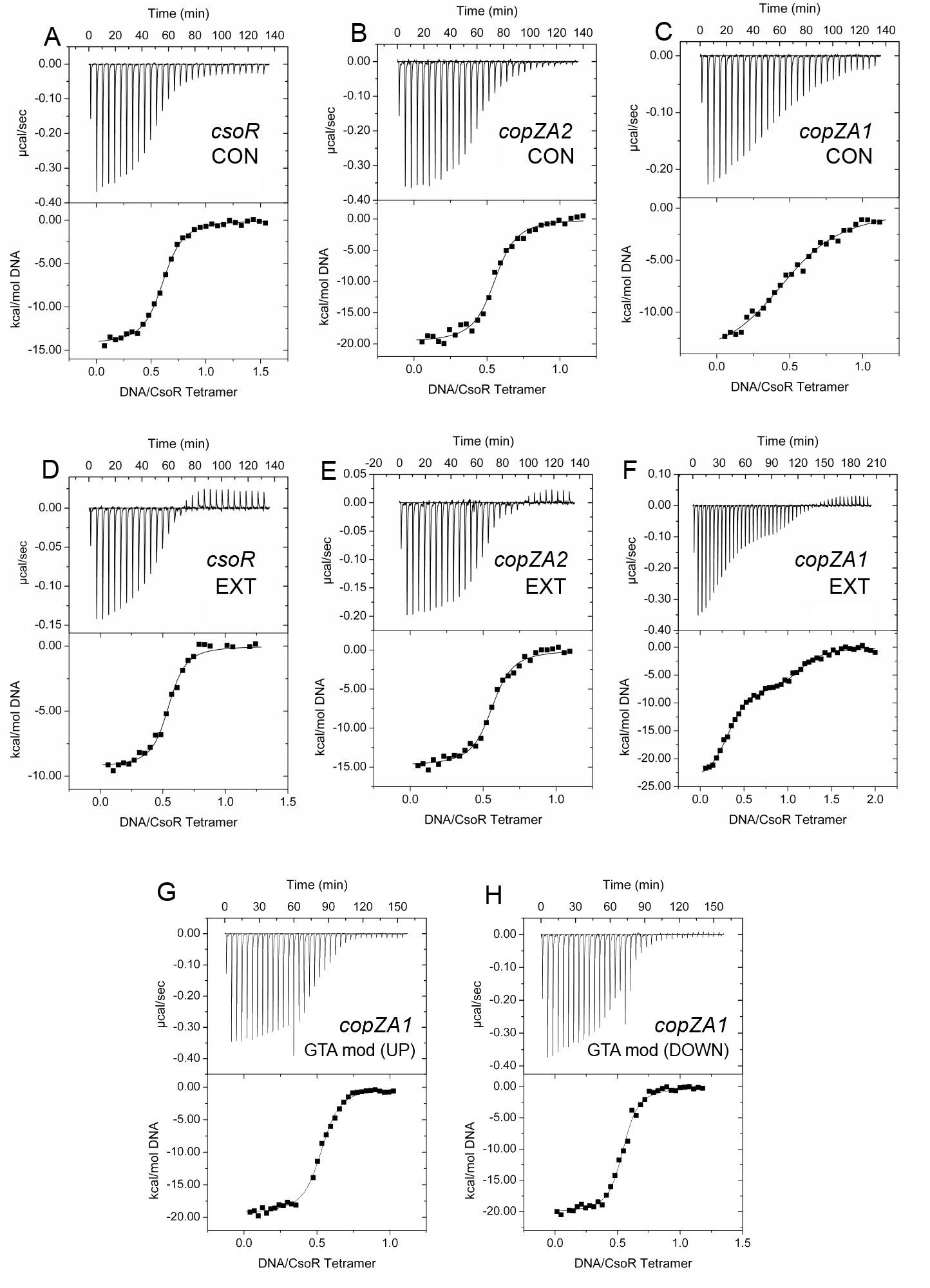


**Figure S4:** Overlay of the reverse titration (Protein into DNA) onto the forward titration (DNA into protein) of the *copZA-*EXT site in 10 mM HEPES, 150 mM NaCl, pH 7.5, 25 °C. Binding parameters for the forward titration are given in the main text and for the forward titration: *N* = 0.86 (± 0.006); *K*_D_ = 112 (± 12) nM; *ΔH_b_* = -15.7 (± 0.16) kcal/mol; *ΔG_b_* = -9.47 (± 0.1) kcal/mol; -*TΔS_b_* = 6.21 (± 0.07) kcal/mol. (n.b. the x-axis label is for the forward titration but is of course CsoR/DNA for the reverse titration).


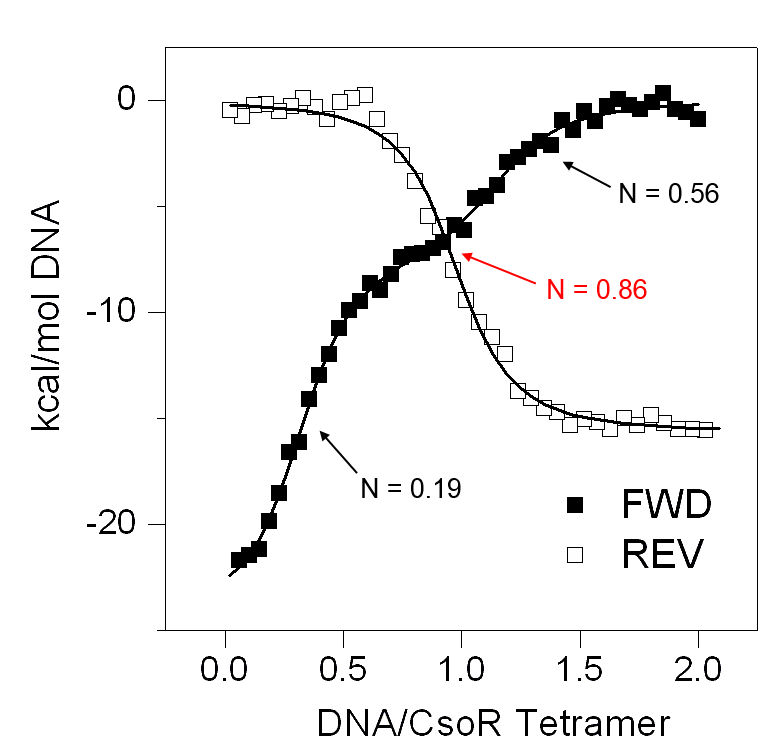


**Figure S5:** Clustal Ω multiple sequence alignment of *E. coli* K12 RcnR against characterised CsoRs, showing the RASK motif of RcnR as compared to the RLXR motif of CsoR proteins in red brackets. Other differences in sequence between RcnR and CsoR include a His residue boxed in red, which in CsoR corresponds to the Cu(I)-binding Cys.


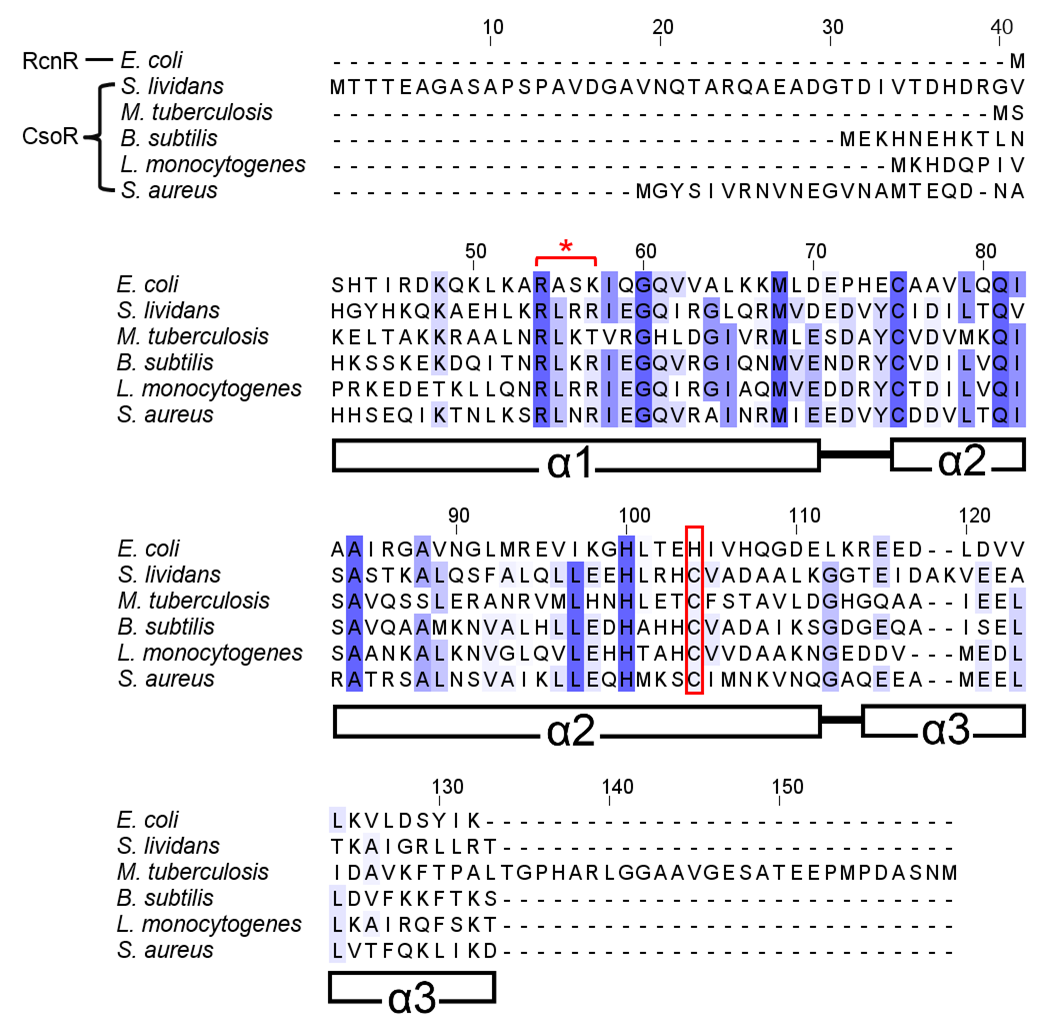

Supplement: Supplementary Data [file supp_gkt902_nar-02414-v-2013-File002.docx]
